# Dry Socket: Incidence, Clinical Features, and Predisposing Factors

**DOI:** 10.1155/2014/796102

**Published:** 2014-06-02

**Authors:** Babatunde O. Akinbami, Thikan Godspower

**Affiliations:** Department of Oral and Maxillofacial Surgery, University of Port Harcourt Teaching Hospital, Rivers State PMB 500004, Nigeria

## Abstract

*Background*. Dry socket is a global phenomenon. The purpose of the study was to investigate the incidence of dry socket in recent times in a Nigerian Tertiary Hospital. *Methods*. Patients who were referred for dental extractions were included in the study. The case files of patients were obtained and information retrieved included biodata, indication for extraction, number and type of teeth extracted, oral hygiene status, compliance to oral hygiene instructions, and development of dry socket. *Results*. One thousand, one hundred and eighty two patients with total of 1362 teeth extracted during the 4-year period of the study were analyzed, out of which 1.4% teeth developed dry socket. The mean age (SD) was 35.2 (16.0) years. Most of the patients who presented with dry socket were in the fourth decade of life. Mandibular teeth were affected more than maxillary teeth. Molars were more affected. Retained roots and third molars were conspicuous in the cases with dry socket. *Conclusion*. The incidence of dry socket in our centre was lower than previous reports. Oral hygiene status, lower teeth, and female gender were significantly associated with development of dry socket. Treatment with normal saline irrigation and ZnO eugenol dressings allowed relief of the symptoms.

## 1. Introduction

Exodontia is the commonest procedure in oral surgery and dentistry [1]. Most patients have to contend with moderate to severe pain over varying periods from not only the indications of these extractions but also the fear of pain from having an extraction which might have been avoided. Occasionally, fears of such patients actually result in real or perceived pain during extraction depending on the skill of the clinician. Some may also have severe pain immediately postoperatively and this may continue for several days after the procedure. Dry socket, also referred to as alveolar or fibrinolytic osteitis, is a major complication that follows extraction of tooth/teeth in oral surgery [2]. It is an acute inflammation of the alveolar bone around the extracted tooth and it is characterized by severe pain, breakdown of the clot formed within the socket making the socket empty (devoid of clot), and often filled with food debris [3]. There is mild swelling and redness of the gingival, halitosis, bone exposure, and severe tenderness on examination.

By the third day postextraction, pain due to extraction is expected to have subsided appreciably, but when such pain becomes worse and continues through one week after the procedure and the socket does not appear to be healing, the occurrence of dry socket can be established. Incidence of dry socket has been reported in literature to be about 0.5–5.6% and following surgical extraction of third molars, it has been found to be up to 30% [4–8]. Several factors have been reported in literature to be responsible for the occurrence of dry socket; these include traumatic, difficult and prolonged extraction, pre- and postoperative infection at the site, smoking, oral contraceptives, bone disorders and underlying pathologies, irradiation, systemic illness such as diabetes mellitus, clotting problems, and failure to comply with postextraction instructions [9–12]. Other possible risk factors include periodontal diseases and previous dry socket with past extractions [13]. This is the first time a research on this disease will be conducted in the 12 years of establishment of our dental center and it will be relevant in order to contribute to existing literature and also to see any recent changing trend. Therefore, the aim of this study was to clinically investigate the incidence of dry socket complicating exodontias in our center.

## 2. Methods

Case files of all patients that attended the dental center and had extractions of their tooth/teeth from January 2010 to December 2013 were obtained from the records department; information retrieved were patients' biodata, oral hygiene status, systemic factors, diagnoses and indications for teeth extraction, teeth extracted, antibiotics prescribed and dosage of antibiotics, compliance to postextraction instructions, and occurrence of dry socket during follow-up. All types of extractions (routine/surgical, retained root/whole tooth/deciduous tooth/impacted tooth) were included. Approval to conduct the research was given by the hospital ethics and research committee. Dry socket was diagnosed based on the presence of severe pain from the socket and the absence of clot in the socket.

Data was fed into the computer; frequencies and proportions were obtained and statistical analysis was done using SPSS software package version 16.00 (SPSS Inc, Chicago, IL, USA). Descriptive statistics included means and standard deviation. Incidence was determined by dividing the number of extractions that presented with dry socket by total number of teeth extracted. Annual incidence and overall 4-year incidence were determined. Relationship between occurrence of dry socket and factors reviewed was determined using regression analysis and *P* values less than 0.05 were considered significant.

## 3. Results

A total of 1182 patients with 1362 extracted teeth were reviewed within the 4-year study, out of which males were 466 (39.4%) and females were 716 (60.6%). Age range was 16–96 years and means (SD) was 35.2 (16.0) years. Patients' attendance was the highest in 2011 (461 (39%)), followed by 2010 (354 (29.9%)) and the least was in 2013 (78 (6.6%)). Male to female ratio for each year is shown (Table 1). Only 29.3% of the cases had systemic diseases. Hypertension was the commonest systemic illness 116 (9.8%) followed by allergies to various drugs and sickle cell disease was the least. Majority (49.0%) of the patients had fair or poor oral hygiene. Only a total of 6% had good oral hygiene while status of the oral hygiene was not stated in a total of 38%. A total of 1052 (89%) patients had extraction of single tooth and 130 (11%) patients had multiple extractions.

Molars constituted the highest number of extracted tooth 1080 (79.3%) with the first molars contributing the highest figure. Lower teeth removed in each year were more than upper teeth. For 2011 and 2012, more right teeth were extracted than left teeth, in contrast to 2010 and 2013. The total of retained roots and impacted teeth extracted in each year was less than 13% for each year. A total of 46 (3.8%) of the extractions were surgical (44 of which involved third molar), 1316 extractions (96.2%) were done by routine method with or without elevators. Figures for compliance to oral hygiene instructions were also reflected (Table 2).

For each year and the whole 4 years, acute apical periodontitis was the commonest indication for extraction 604 (44.4%), followed by irreversible pulpitis 162 (11.9%). Failed root canal treatments, cervical lesions, tooth displacements/malposition, periodontal abscess, and chronic apical periodontitis (apical abscess, granuloma, and cysts) were among the least indications. Incidence of dry socket for each year was 2.4%, 1.1%, 0.6%, and 1.0%, respectively, and overall 4 year incidence was 1.4% (Table 3).

**Figure 1:**
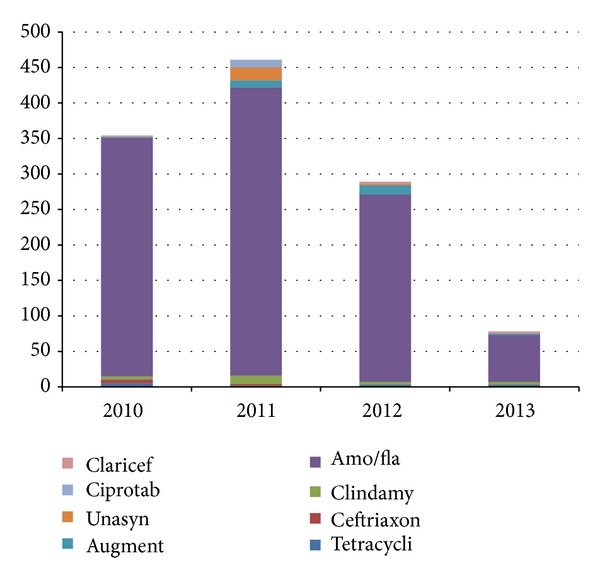
Annual distribution of antibiotics used.

Antibiotics were routinely prescribed following all extractions; on the whole and for each year, the combination of amoxicillin (500 mg 8 hrly and metronidazole 400 mg 8 hrly for 5 days) constituted the highest figure followed by amoxicillin/clavulanic acid (Augmentin 625 mg 8 hrly for 5 days) (Figure 1).

A total of 19 patients had dry socket (1.4%) (Table 4). More female patients had dry socket than males (36.8%) but no significant relationship with dry socket, *P* > 0.05, 0.393, and most of the patients (47.4%) were in the fourth decade. There was significant relationship between fair/poor oral hygiene with dry socket, *P* < 0.05, and 0.035. A total of 14 (73.7) patients had nonsurgical extractions and most of these also involved the lower molars, with significant relationship, *P* < 0.05, 0.013. The side distribution was more on the right, 11 (57.8%). Also, there was almost equal distribution of indications for exodontias amongst the cases with no strong relationship with any of the reasons. Seven (36.8%) patients with dry socket did not comply with oral hygiene instruction regarding the thorough use of warm salt mouth bath. Same number of patients did not comply and they also had dry socket, but in 5 cases with dry socket, compliance was not stated. Alternate day normal saline irrigation and ZnO eugenol dressings were our mainstay of treatment.

## 4. Discussion

The exact etiology and mechanism of dry socket are not exactly known but several factors have been associated. Careful analysis into the pathophysiology of dry socket (DS) stated that poor oral hygiene, vasoconstrictors, and reduced blood supply are important factors but reports have placed emphasis on trauma from difficult exodontias causing fibrinolysis and release of pain inducing chemical substances [14, 15].

There were more females (63.2%) that presented with dry socket than males and most of the patients were in the fourth decade; these findings corroborate other reports [3, 16, 17] but in Lagos [17], the ratio gap was much higher, 1 : 4.4, and age was more in third decade. Eighty-nine percent had extraction of single tooth and this was similar to the study of Upadhyaya and Humagain [16]. Reasons may be hormonal, coupled with the use of contraceptives by some women which is another major factor; but such histories were not retrieved and we could not ascertain a relationship of dry socket with such drugs; however, one hypertensive, 1 pregnant patient, and 2 cases of peptic ulcer disease had dry socket but there was no strong link with these diseases. No patient with diabetes mellitus had dry socket in our study in contrast to few other reports [1, 15].

Also, there were more in mandibular teeth (68.4%) than maxillary teeth and this was similar to other studies [16–18]. Dry socket occurred in only 2 cases with multiple extractions involving two and three teeth; the specific tooth/teeth involved were not specified but it was notable that in both cases, all the five teeth removed were retained roots. In addition, amongst cases of dry socket, last molars were more involved. There were no cases of dry socket from exodontias of deciduous teeth and all these supported the fact that difficult extraction which was experienced with most retained roots and some last molars is a major contributor to dry socket [14, 18].

Overall incidence in this study was 1.4% and much less than figures documented in most reports outside Nigeria and the 5.6% in the study of Houston et al. [14–19]. Relationship of dry socket was statistically significant with lower teeth and oral hygiene. Removal of debris is poorer in lower sockets than upper teeth and this may be contributory. Of the total cases of dry socket, only 36.8% were noncompliant with oral hygiene instructions; information was not available from other studies on compliance to oral hygiene instructions.

One major factor that has been documented in literature that predisposes to dry socket is smoking [20]; avoidance of smoking within the period of healing is a component of the postextraction instructions, but the level of compliance to such specific instruction was ambiguous, again; pre-extraction plasma/tissue levels of nicotine and other nitrous amines might also possibly enhance the occurrence of dry socket; in this study, the smoking status of most of the patients with dry socket was not directly stated but almost all had a fair or poor oral hygiene. In the study from Lagos, 11.1% of those with dry socket were smokers [16], and we also recorded 10.5%.

Acute apical periodontitis was the commonest indication for exodontias; this was closely similar to figures from other reports [7, 13]. Indication for extraction was not stated in about 13% of cases and this involved the cases with multiple teeth; this was probably due to the fact that it was only one main tooth that was causing severe pain that brought patient to the hospital. Such pains are commonly due to acute pulpitis/irreversible pulpitis, acute apical periodontitis, and dentoalveolar abscesses. Other teeth indicated for extraction were incidental probably due to the mobility of tooth/teeth from chronic periodontitis or grossly carious painless teeth with pulp necrosis.

Operator technique and skill are essential factors in the occurrence of dry socket [11, 16]; however, we could not evaluate this very important factor because of the retrospective nature of the study; In our center, surgical exodontias are usually performed by resident doctors and routine exodontias are performed by house officers or final year students under supervision of the consultants or residents, and considering the low incidences from this study, it may be deduced that appropriate techniques were utilized for these procedures to a large extent. Antibiotics were routinely given to all patients following exodontias in our center and this probably may have contributed to the low incidences. We used mostly amoxicillin and metronidazole followed by amoxicillin/clavunate and clindamycin. Most mixed infections are susceptible to these antibiotics and systematic reviews have proved that prophylactic antibiotics and chlorhexidine (0.12% or 0.2%) rinses or gel (0.2%) in the sockets of extracted teeth minimized dry socket, but use of Surgicel gauze pack has been found to increase the incidence [21–25].

In conclusion, acute apical periodontitis was the highest reason for exodontias in our study. Our overall incidence was 1.4%. The factors associated with dry socket were lower teeth, molars, female gender, and patients with inadequate oral hygiene. We had a larger sample size and our study reflected lower annual incidences compared to earlier studies in literature and this might be related to emphasis placed on meticulous and appropriate techniques of extraction, use of antibiotics and compliance to oral hygiene instructions.

## Figures and Tables

**Table 1:** Oral hygiene status and systemic factors in 1182 patients.

	2010 *n* (%)	2011 *n* (%)	2012 *n* (%)	2013 *n* (%)	Total
M : F ratio	1 : 1.5	1 : 1.5	1 : 1.7	1 : 1.4	1 : 1.5
Oral hygiene					
Poor	109 (30.8)	153 (23.9)	88 (30.4)	46 (59.0)	396 (33.5)
Fair	141 (39.8)	254 (55.1)	160 (55.4)	24 (30.8)	579 (49.0)
Good	19 (5.4)	29 (6.3)	16 (5.5)	8 (10.2)	72 (6.1)
Not stated	85 (24.0)	24 (5.2)	25 (8.7)	0 (0)	134 (11.3)
Multiple teeth	41 (11.6)	53 (11.5)	32 (11.1)	4 (5.1)	130 (11.0)
Single tooth	313 (88.4)	408 (88.5)	257 (88.9)	74 (94.9)	1052 (89.0)
Systemic factors					
Hypertension	32 (9.0)	44 (9.5)	37 (12.8)	3 (3.8)	116 (9.8)
Diabetics	6 (1.7)	9 (2.0)	9 (3.1)	5 (6.4)	29 (2.5)
Sickle cell disease	0 (0)	1 (0.2)	0 (0)	0 (0)	1 (<0.1)
Pregnancy	9 (2.5)	10 (2.2)	4 (1.4)	0 (0)	23 (1.9)
Peptic ulcer disease	11 (3.1)	16 (3.5)	25 (8.7)	3 (3.8)	55 (4.7)
Allergy	6 (1.7)	44 (9.5)	48 (16.6)	5 (6.4)	103 (8.7)
Asthma	2 (0.9)	15 (3.3)	2 (0.7)	0 (0)	19 (1.6)
Total	66 (18.6)	139 (30.2)	125 (43.3)	16 (20.4)	346 (29.3)
Total number of patients	**354 (29.9)**	**461 (39.0)**	**289 (24.5)**	**78 (6.6)**	**1182 (100)**

**Table 2:** Local factors and instruction compliance level in 1182 patients with 1362 exodontia.

	2010 *n* (%)	2011 *n* (%)	2012 *n* (%)	2013 *n* (%)	Total
Local factors					
Type & site of tooth					
Anterior	21 (5.1)	49 (9.6)	25 (7.8)	12 (11.5)	107 (7.9)
Premolars	51 (12.3)	71 (13.6)	46 (14.4)	7 (6.7)	175 (9.4)
Molars	343 (82.6)	403 (76.8)	249 (77.2)	85 (82.8)	1080 (79.3)
Permanent	412 (99.3)	521 (99.6)	314 (98.1)	103 (99.0)	1350 (99.1)
Deciduous	3 (0.7)	2 (0.4)	6 (1.9)	1 (1)	12 (0.9)
Upper	178 (42.9)	128 (25.0)	150 (46.9)	28 (26.9)	484 (35.5)
Lower	237 (57.1)	395 (75.0)	170 (53.1)	76 (73.1)	878 (64.5)
Right	209 (50.4)	287 (55.9)	175 (54.7)	41 (39.4)	712 (52.3)
Left	206 (49.6)	236 (44.1)	145 (45.3)	63 (60.1)	650 (47.7)
Retained root	26 (6.3)	33 (6.3)	22 (6.9)	4 (3.8)	85 (6.2)
Impacted tooth	19 (4.6)	14 (2.7)	5 (1.6)	3 (2.9)	41 (3.0)
Erect tooth	370 (89.1)	476 (91.0)	293 (91.5)	97 (93.3)	1056 (90.8)
Mode of exodontias					
Routine	394 (94.9)	513 (98.1)	314 (98.1)	95 (91.3)	1316 (99.2)
Surgical	21 (5.1)	10 (1.9)	6 (1.9)	9 (8.7)	46 (0.8)
Total number of teeth	**415 (30.5)**	**523 (38.4)**	**320 (23.5)**	**104 (7.6)**	**1362 (100)**
Oral hygiene instruction					
Compliant	61 (17.2)	114 (24.7)	71 (24.6)	24 (30.8)	270 (23)
Noncompliant	4 (1.1)	3 (0.7)	3 (1.0)	1 (1.3)	11 (1)
Not stated	289 (82.7)	344 (74.6)	215 (74.4)	53 (67.9)	901 (76)
Total number of patients	**354** (**29.9**)	**461 (39.0)**	**289 (24.5)**	**78 (6.6)**	**1182 (100)**

**Table 3:** Indications of exodontia and incidence of dry socket in 1182 patients with 1362 teeth.

Indication for exodontia	2010 *n* (%)	2011 *n* (%)	2012 *n* (%)	2013 *n* (%)	Total
Reversible pulpitis	21 (5.1)	15 (2.9)	2 (0.6)	2 (6.7)	40 (2.9)
Irreversible pulpitis	63 (15.2)	47 (9.0)	40 (12.5)	12 (11.5)	162 (11.9)
Retained root	26 (6.3)	33 (6.3)	22 (6.9)	4 (3.8)	85 (6.2)
Pericoronitis	19 (4.6)	14 (2.7)	5 (1.6)	3 (2.9)	41 (3.0)
Chronic apical periodontitis	8 (1.9)	8 (1.5)	1 (0.3)	3 (2.9)	20 (1.5)
Acute apical periodontitis	148 (35.7)	261 (49.9)	163 (50.9)	32 (30.8)	604 (44.3)
Chronic periodontitis	25 (6.0)	22 (4.2)	14 (4.4)	7 (6.7)	68 (5.0)
Dentoalveolar abscess	23 (5.5)	28 (5.4)	21 (6.6)	6 (5.8)	78 (5.7)
Periodontal abscess	4 (1.0)	3 (0.6)	1 (0.3)	1 (1.0)	9 (0.7)
Fracture	15 (3.6)	27 (5.2)	9 (2.8)	5 (4.8)	56 (4.1)
Cervical abrasion	0 (0)	1 (0.2)	2 (0.6)	1 (1.0)	4 (0.3)
Failed RCT	2 (0.5)	1 (0.2)	2 (0.6)	0 (0)	5 (0.4)
Displacement	0 (0)	1 (0.2)	7 (2.2)	0 (0)	8 (0.8)
Not stated	61 (14.8)	62 (11.9)	31 (9.7)	27 (26.0)	181 (13.3)
Incidence rate					
Number of teeth with dry socket	10 (2.4)	6 (1.1)	2 (0.6)	1 (1.0)	19 (1.4)
Total number of teeth	415 (30.5)	523 (38.4)	320 (23.5)	104 (7.6)	1362 (100)
Incidence	2.4%	1.1%	0.6%	1.0%	1.4%

**Table 4:** Demographics and characteristics of 19 patients with dry socket.

S/number	Age/sex	Oral hygiene status	Systemic illness	Tooth	Indication	Mode	Antibiotic	Oral hygiene instruction
1	F/45	Poor	None	Upper Rt 8	Impacted	Routine	Zinnat/Diclofenac	Not stated
2	F/47	Fair	Hypertensive	Upper Rt 4	Supernumerary	Routine	amoxyl/flagyl	Noncompliant
3	M/22	Fair, smoke, alcohol	None	Lower Lt 8	Pericoronitis	Surgical	amoxyl/flagyl	Compliant
4	M/33	Fair	None	Lower Lt 6	Irreversible pulpitis	Routine	amoxyl/flagyl	Not stated
5	F/45	Poor	None	Lower Lt 8	Irreversible Pulpitis	Surgical	amoxyl/flagyl	Not stated
6	F/35	Not stated, alcohol	None	Lower Lt 8	Impacted	Surgical	amoxyl/flagyl	Noncompliant
7	M/30	Fair	None	Lower Rt 7	Irreversible pulpitis	Surgical	amoxyl/flagyl	Not stated
8	F/30	Fair	Pregnant	Lower Rt 6	Acute apical periodontitis	Routine	amoxyl/flagyl	Compliant
9	F/45	Fair	None	Upper Rt 8 and Lt 5	Retained roots	Routine	amoxyl/flagyl	Not stated
10	M/35	Fair, smoke, alcohol	None	Lower Lt 6	Dentoalveolar abscess	Routine	Clindamycin	Compliant
11	F/37	Fair	None	Lower Rt 6	Acute apical periodontitis	Routine	amoxyl/flagyl	Noncompliant
12	F/57	Fair	None	Upper Rt 87 and Lt 6	Retained roots	Routine	unasyn/flagyl	Compliant
13	M/30	Fair	None	Lower Lt 8	Irreversible pulpitis	Surgical	amoxyl/flagyl	Not compliant
14	M/62	Poor	None	Lower Lt 5	Dentoalveolar abscess	Routine	amoxyl/flagyl	Compliant
15	F/18	Not stated	None	Lower Rt 7	Irreversible pulpitis	Routine	amoxyl/flagyl	Compliant
16	F/31	Fair	None	Lower Rt 6	Acute periodontitis	Routine	Clindamycin	Compliant
17	M/26	Fair	Peptic ulcer disease	Lower Lt 8	Acute apical periodontitis	Routine	amoxyl/flagyl	Noncompliant
18	F/28	Good	Peptic ulcer disease	Upper Rt 8	Acute apical periodontitis	Routine	amoxyl/flagyl	NonCompliant
19	F/34	Fair	None	Upper Rt 8	Reversible pulpitis	Routine	amoxyl/flagyl	Not compliant

## References

[1] Nusair YM, Younis MH (2007). Prevalence, clinical picture, and risk factors of dry socket in a Jordanian Dental Teaching Center. *Journal of Contemporary Dental Practice*.

[2] Momeni H, Shahnaseri S, Hamzeheil Z (2011). Evaluation of relative distribution and risk factors in patients with dry socket referring to Yazd dental clinics. *Dental Research Journal*.

[3] Cohen ME, Simecek JW (1995). Effects of gender-related factors on the incidence of localized alveolar osteitis. *Oral Surgery, Oral Medicine, Oral Pathology, Oral Radiology and*.

[4] Swanson AE (1990). Prevention of dry socket: an overview. *Oral Surgery Oral Medicine and Oral Pathology*.

[5] Vezeau PJ (2000). Dental extraction wound management: medicating postextraction sockets. *Journal of Oral and Maxillofacial Surgery*.

[6] Oginni FO, Fatusi OA, Alagbe AO (2003). A clinical evaluation of dry socket in a Nigerian teaching hospital. *Journal of Oral and Maxillofacial Surgery*.

[7] Jaafar N, Nor GM (2000). The prevalence of post-extraction complications in an outpatient dental clinic in Kuala Lumpur Malaysia—a retrospective survey. *Singapore Dental Journal*.

[8] Khawaja NA (2006). Incidence of dry socket in lower jaw at a teaching dental hospital. *Pakistan Oral and Dental Journal*.

[9] Blum IR (2002). Contemporary views on dry socket (alveolar osteitis): a clinical appraisal of standardization, aetiopathogenesis and management: a critical review. *International Journal of Oral and Maxillofacial Surgery*.

[10] Adeyemo WL, Ladeinde AL, Ogunlewe MO (2006). Clinical evaluation of post-extraction site wound healing. *Journal of Contemporary Dental Practice*.

[11] Sisk AL, Hammer WB, Shelton DW, Joy ED (1986). Complications following removal of impacted third molars: the role of the experience of the surgeon. *Journal of Oral and Maxillofacial Surgery*.

[12] Betts NJ, Makowski G, Shen Y-H, Hersh EV (1995). Evaluation of topical viscous 2% lidocaine jelly as an adjunct during the management of alveolar osteitis. *Journal of Oral and Maxillofacial Surgery*.

[13] Cheung LK, Chow LK, Tsang MH, Tung LK (2001). An evaluation of complications following dental extractions using either sterile or clean gloves. *International Journal of Oral and Maxillofacial Surgery*.

[14] Masuck R, Klammt J (1991). The role of fibrinolysis in the pathogenesis of alveolitis after tooth extraction: preliminary report. *Deutsche Stomatologie*.

[15] Houston JP, McCollum J, Pietz D, Schneck D (2002). Alveolar osteitis: a review of its etiology, prevention, and treatment modalities. *General Dentistry*.

[16] Upadhyaya C, Humagain M (2010). Prevalence of dry socket following extraction of permanent teeth at kathmandu university teaching hospital (KUTH), Dhulikhel, Kavre, Nepal: a study. *Kathmandu University Medical Journal*.

[17] Ogunlewe MO, Adeyemo WL, Ladeinde AL, Taiwo OA (2007). Incidence and pattern of presentation of dry socket following non-surgical tooth extraction. *Nigerian Quarterly Journal of Hospital Medicine*.

[18] Amler MH (1999). Disturbed healing of extraction wounds. *The Journal of Oral Implantology*.

[19] Meechan JG, Macgregor IDM, Rogers SN, Hobson RS, Bate JPC, Dennison M (1988). The effect of smoking on immediate post-extraction socket filling with blood and on the incidence of painful socket. *British Journal of Oral and Maxillofacial Surgery*.

[20] Field EA, Speechley JA, Rotter E, Scott J (1985). Dry socket incidence compared after a 12 year interval. *British Journal of Oral and Maxillofacial Surgery*.

[21] Shepherd J (2005). Rinsing with chlorhexidine may reduce dry socket after third molar surgery. *Oral Surgery, Oral Medicine, Oral Pathology, Oral Radiology and Endodontology*.

[22] Chapnick P, Diamond LH (1992). A review of dry socket: a double-blind study on the effectiveness of clindamycin in reducing the incidence of dry socket. *Journal of the Canadian Dental Association*.

[23] Alexander RE (2000). Dental extraction wound management: a case against medicating postextraction sockets. *Journal of Oral and Maxillofacial Surgery*.

[24] Adeyemo WL, Ladeinde AL, Ogunlewe MO (2007). Influence of trans-operative complications on socket healing following dental extractions. *Journal of Contemporary Dental Practice*.

[25] Garibaldi JA, Greenlaw J, Choi J, Fotovatjah M (1995). Treatment of post-operative pain. *Journal of the California Dental Association*.

